# Significance of PD-L1 and Tumor Microenvironment in Laryngeal Squamous Cell Cancer

**DOI:** 10.3390/cancers16152645

**Published:** 2024-07-25

**Authors:** Filip Tudor, Blažen Marijić, Emina Babarović, Ita Hadžisejdić

**Affiliations:** 1 Department of Otorhinolaryngology, Head and Neck Surgery, Clinical Hospital Center Rijeka, 51000 Rijeka, Croatia; filip.tudor@uniri.hr (F.T.); blazen.marijic@uniri.hr (B.M.); 2 Faculty of Medicine, University of Rijeka, 51000 Rijeka, Croatia; emina.babarovic@uniri.hr; 3 Clinical Department of Pathology and Cytology, Clinical Hospital Center Rijeka, 51000 Rijeka, Croatia

**Keywords:** laryngeal carcinoma, PD-L1, tumor microenvironment, survival

## Abstract

**Simple Summary:**

Advanced stage laryngeal squamous cell cancer (LSCC) continues to have poor prognosis, with 5-year survival from 50 to 60% and poor functional outcome. PD-L1 and tumor microenvironment (CD4, CD8, CD68 and CD163) expression were investigated in LSCC using immunohistochemistry. PD-L1 expression showed statistically significant positive correlation with all examined tumor microenvironment cells. Higher CD68 and CD163 expression represented significantly worse prognosticators for clinical outcome in patients with LSCC. To find out which LSCC patients will gain from immunomodulation therapies, it is important to understand the relationship between PD-L1 expression, immune cell distribution and prognosis in LSCC patients.

**Abstract:**

Background: Despite the considerable advancement in the field of medicine over recent decades, laryngeal cancer continues to be a challenge. The field of immune oncology has generated promising immunomodulation therapies and opened up new ways of treatment. Methods: Our retrospective study included 102 patients diagnosed with laryngeal squamous cell cancer (LSCC). Immunohistochemistry was used to evaluate the expression of PD-L1 and tumor microenvironment cells (CD4, CD8, CD68 and CD163). Results: PD-L1 expression showed statistically significant positive correlations with all examined tumor microenvironment cells. Patients with high CD68 and CD163 expression intratumorally (*p* = 0.0005 and *p* = 0.006, respectively) had statistically significant shorter disease-specific survival. Moreover, a statistically shorter time to recurrence was found in patients with high CD68 intratumoral and CD8 overall counts (*p* = 0.049 and *p* = 0.019, respectively). Also, high CD8 overall (>23%) and CD68 intratumoral (>2.7%) expression were statistically significant predictors of recurrence (*p* = 0.028, OR = 3.11 and *p* = 0.019, OR = 3.13, respectively). Conclusions: Higher CD68 and CD163 expression represented significantly worse prognosticators for clinical outcomes in patients with LSCC. In order to determine which LSCC patients will benefit from anti-PD-1/PD-L1 inhibitors, it is crucial to elucidate the relationship between PD-L1 expression, immune cell distribution and prognosis in LSCC patients.

## 1. Introduction

Despite the considerable advancement in the field of medicine over the recent decades, laryngeal cancer continues to be a challenge. Patients in the early stage of disease exhibit an excellent oncological prognosis, in contrast to the patients with advanced stage who have a five-year survival rate ranging from 50% to 60% and an unfavorable functional outcome [1]. The proportion of patients classified as advanced stage at the time of diagnosis ranges from approximately 60% to 75%. As opposed to the oncological outcomes observed in other tumor subsites of the head and neck, 5-year survival rates for laryngeal cancer have decreased during the past three decades [2,3]. 

In the numerous subsites of head and neck squamous cell cancer (HNSCC), including the larynx, the field of immune oncology has generated promising immunomodulation therapies and opened up new ways of treatment. Currently, the use of checkpoint inhibitors that target the programmed cell death ligand pathway (PD-L) are primarily restricted to the cases of recurrent, persistent or metastatic disease in HNSCC. These inhibitors have shown potential as therapeutic targets in various types of cancer, emphasizing the significance of the immune response.

The existing body of research relating to the laryngeal cancer and PD-L1 exhibits inconsistent findings. Several studies have demonstrated that increased expression of PD-L1 correlates with improved immune response, as well as statistically significant increase in disease-free and overall survival [4,5]. Conversely, other studies have indicated that elevated levels of PD-L1 are linked to a higher incidence of distant metastases [6,7]. Furthermore, investigation of the tumor microenvironment has garnered attention due to its potential to have prognostic and therapeutic implications [8]. The tumor microenvironment is a complex cell ecology that coexists with and supports tumor cells as they progress toward malignancy. Innate and adaptive immune cells alongside macrophages are drawn to the tumor site, and they may be seen at any stage of tumor growth. The expression of PD-L1 is dependent upon a multifaceted interplay between tumor cells and immune cells within the tumor microenvironment. The presence of IFN-γ, which is secreted by T lymphocytes, serves to augment the expression of PD-L1 in tumor cells [4]. In this context, the upregulation of PD-L1 in neoplastic cells may be attributed to the immunological response exerted by tumor-infiltrating lymphocytes, which possess antitumor properties. If this is the case, it is probable that individuals with T lymphocyte-rich tumors exhibiting a high level of PD-L1 expression will experience a heightened immune response, potentially leading to a more favorable prognosis [4,9]. Also, tumor-associated macrophages (TAMs) seem to play an important role in tumor growth and therapeutic responses. TAMs consist of both M1 macrophages, which promote antitumor immunity, and M2 macrophages, which have pro-tumorigenic features [10]. Tumor-associated macrophages (TAMs) facilitate the evasion of immune responses by attracting immunosuppressive cells such as regulatory T cells and promoting angiogenesis [11]. Researchers have shown a correlation between increased frequencies of TAMs and worse outcomes in several types of malignancies [10]. However, there are not many studies investigating TAMs in laryngeal squamous cell cancer (LSCC). Moreover, there is lack of studies that investigate the distribution of immune cells (stromal vs. intratumoral), as well as compare the correlation between TAMs and PD-L1 and other immune cells of the tumor microenvironment, which possibly could be considered as a predictive factor for immune therapies. Therefore, the aims of our study are (i) to assess the immune cell quantity and distribution, mainly for CD4, CD8, CD68 and CD163 in LSCC; (ii) to examine the correlation between PD-L1 expression and the tumoral immune microenvironment (CD4, CD8, CD68 and CD163) in LSCC; (iii) to correlate the pathological and clinical features of LSCC with PD-L1 expression; (iv) to determine PD-L1 expression and tumoral microenvironment distribution with disease outcome in patients with LSCC.

## 2. Materials and Methods

### 2.1. Study Cohort

This retrospective study was conducted using samples obtained from the archives of the Clinical Department of Pathology and Cytology, Clinical Hospital Center Rijeka. The biopsy samples included in this study were obtained from the patients that were treated for laryngeal neoplasms at the Clinic for Otorhinolaryngology and Head and Neck Surgery, Clinical Hospital Center Rijeka, between 2010 and 2019. The study was approved by the Institutional Review Board of Clinical Hospital Center Rijeka, Croatia (No. 2170-29-02/1-22-2), with annual extensions. All the patients were primarily surgically treated. After the surgery, depending on the extent of the disease, pathohistological findings and surgical margins, the patients were further treated with radio- or chemoradiotherapy. The cohort included 102 patients diagnosed with laryngeal squamous cell cancer (LSCC). Furthermore, patients that had prior oncological treatment, such as radiation or chemotherapy, or patients who had malignancies of the oropharynx, hypopharynx or other primary tumors, were not included in the study. Information such as the patient’s age at the time of the first diagnosis, the patient’s consumption of alcohol and smoking, the size of the tumor, the presence of lymph node metastases, lymphovascular and perineural invasion and the TNM stage were obtained from the patient’s medical records. The eighth iteration of the AJCC/UICC TNM staging system was utilized for the clinical staging [12]. According to the preliminary PD-L1 expressions in LSCC patients, we calculated the sample size (total sample size *n* = 92) for the sample size comparison of proportions at the level of statistical significance. *p* < 0.05 with a statistical analysis power of 80% using options “Sample size calculation” in MedCalc for Windows, version 19.1 (MedCalc Statistical Software bvba, Ostend, Belgium).

### 2.2. Immunohistochemistry 

The tissue microarrays (TMAs) were constructed using three or four 1 mm cores of the above-mentioned archived biopsy samples. Also, to compensate the spatial distribution of the examined markers, we used serial sections of the same TMA cores. During the immunohistochemical procedures, some cores were either lost, fragmented or showed suboptimal staining; therefore, the number of examined samples sometimes differed between analyses. The antibodies used in this research were as follows: (i) for PD-L1 (mouse monoclonal antibody (IgG) anti-PD-L1 (clone SP263, Ventana, Tucson, SAD)); (ii) for CD4 (mouse monoclonal antibody (IgG) anti-CD4 (clone SP35, Cell Marque, Rocklin, SAD)); (iii) for CD8 (mouse monoclonal antibody (IgG1) anti-CD8 (clone C8/144B, DakoAgilent, Santa Clara, SAD)); (iv) for CD 68 (mouse monoclonal antibody (IgG) anti-CD68 (clone PG-M1, DakoAgilent, Santa Clara, SAD)); (v) for CD163 (mouse monoclonal antibody (IgG1) anti-CD163 (clone 10D6, Leica Biosystems, Buffalo Grove, SAD)). The antigen retrieval protocol, incubation and other procedural steps included in the immunohistochemistry technique for the sample preparation were conducted in accordance with the guidelines provided by the manufacturer. CD4, CD8, CD68 and CD163 were used for tumor microenvironment expression due to their well-known function in the immune system and tumor development. In cancer surveillance, the CD4+ and CD8+ lymphocytes perform a crucial role in eliminating cancerous cells. CD8 lymphocytes stands out due to their antitumor properties, which, when increased, lead to better outcomes in different types of cancer [13,14,15]. CD68 is the most common marker for all macrophages, while CD163 is the most widely used marker for M2 polarized macrophages.

### 2.3. Evaluation of Immunoreactivity

The independent evaluation of the expression of the investigated biomarkers was conducted by two pathologists who were blinded to the patients’ follow-up data. The combined positive score (CPS) and tumor proportion score (TPS) were used to assess the level of PD-L1 expression, where CPS < 1 and TPS < 1 indicated negative expression and CPS ≥ 1 and TPS ≥ 1 indicated positive expression. The CPS was calculated as follows: the number of PD-L1-positive cells, including tumor cells, macrophages and lymphocytes, was divided by the total number of viable tumor cells and then multiplied by 100 [16]. TPS was calculated by the number of PD-L1-positive tumor cells divided by the total number of all viable tumor cells and then multiplied by 100 [17]. TMA cores that contained less than 100 viable tumor cells were excluded.

**Figure 1:**
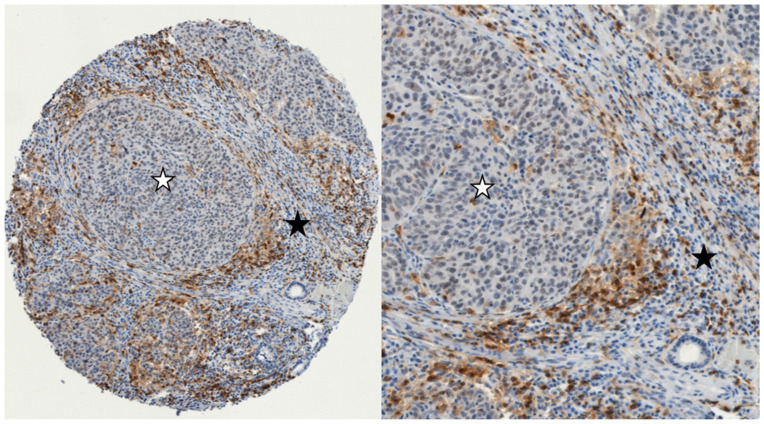
Example of immunohistochemistry expression of CD4+ cells in two different compartments in LSCC: The intratumoral epithelial compartment with iCD4+ cells (tumor cell nests marked with a white asterisk), and intratumoral stromal compartment with sCD4+ cells (marked with a black asterisk). (**left**): 100× magnification; (**right**): 300× magnification (iCD4+: intratumoral; sCD4+: stromal).

The assessment methodology used for CD4, CD8, CD68 and CD163 was derived from the Guidelines for the Assessment of Tumor-Infiltrating Lymphocytes (TILs) in Solid Tumors: Recommendations by an International Immuno-Oncology Biomarker Working Group [18,19]. The evaluation included the assessment of immunocompetent cells at a magnification of 200× in two distinct regions: the intratumoral epithelial compartment, which consists of tumor cell nests, and the tumor stromal compartment, which refers to the tissue located between cancer cell nests inside the tumor. The average density of certain cells was quantified as a continuous variable by calculating the proportion of the area occupied by immunohistochemically positive cell infiltrates in a specific compartment (either tumor cell nests or tumor stroma) relative to the total intratumoral or tumor stromal area. For example, the intratumoral percentage of CD4 cells was determined by dividing the area occupied by CD4 cells in tumor cell nests by the total area of tumor cell nests (Figure 1). Additionally, the density of certain cells was calculated by the number of overall positive cells in a whole specimen relative to the number of all viable tumor cells. A comprehensive evaluation of the tumor region was conducted on each slide, with the exclusion of regions exhibiting ulceration and necrosis from the analysis.

### 2.4. Statistical Analysis

The statistical analysis was conducted using MedCalc for Windows, version 19.1 (MedCalc Statistical Software bvba in Ostend, Belgium). Frequency differences of the nominal variables were assessed using Fisher’s exact test and the chi-square test. Spearman’s rank correlation analysis was used to determine the association between PD-L1 and immune cells. The analysis of tumor recurrence prediction was done using logistic regression. The Kaplan–Meier method was used to compute the cumulative survival probability. The disparities in survival rates were assessed using a log-rank test. All tests conducted were two-tailed, and a statistically significant result was defined as *p* < 0.05.

A receiver operating characteristic (ROC) curve was generated to evaluate the efficacy of CD4, CD8, CD68 and CD163 (intratumoral, stromal and overall) as biomarkers for predicting patient outcomes and determining the most effective statistical cut-off values. Hence, the ROC curve and Youden index were computed to optimize the sensitivity and specificity of the individual marker in predicting the overall disease-specific survival in the univariate model. The AUC (area under the ROC curve) was calculated to assess the prediction model’s quality, along with a 95% confidence interval (CI). ROC analysis showed statistically significant cut-off values of >2.7% for CD68 intratumoral (*p* = 0.004, AUC = 0.702), >5.5% for CD68 overall (*p* = 0.042, AUC = 0.644) and >2% for CD163 intratumoral (*p* = 0.01, AUC = 0.689). Disease-specific survival (DSS) was expressed as the number of months from diagnosis to the occurrence of a disease-related death. Disease-free survival (DFS) was defined as the time interval from the date of diagnosis to the date of the first documented recurrence of disease. If there was no recurrence, disease-free survival was determined as the date of last follow-up.

## 3. Results

### 3.1. Study Cohort

The study comprised 102 LSCC patients, 95 male and 7 female, while the median age was 63 years (range 43.9–83.6 years). The majority of tumors were graded as well or moderately differentiated (G1: 21.4% and G2: 54.4%), and there was 18.4% poorly differentiated (G3) tumors. Also, the LSCC group consisted of 26 (25.5%) T1, 17 (16.7%) T2, 40 (39.2%) T3 and 19 (18,6%) T4 tumors, while positive neck nodes were found in 15 (14.7%) patients. Subsequently, 44 (43.1%) patients were classified as early (stage I and II) and 58 (56.8%) patients as advanced stage disease (stage III and IV). Postoperative radiotherapy was applied in 54 (52.9%) patients. Table 1 shows in more detail the demographic features of the LSCC group.

### 3.2. Expression of PD-L1, CD4, CD8, CD68 and CD163 in LSCC and Comparison between the Early and Advanced Stages of Carcinoma

**Figure 2:**
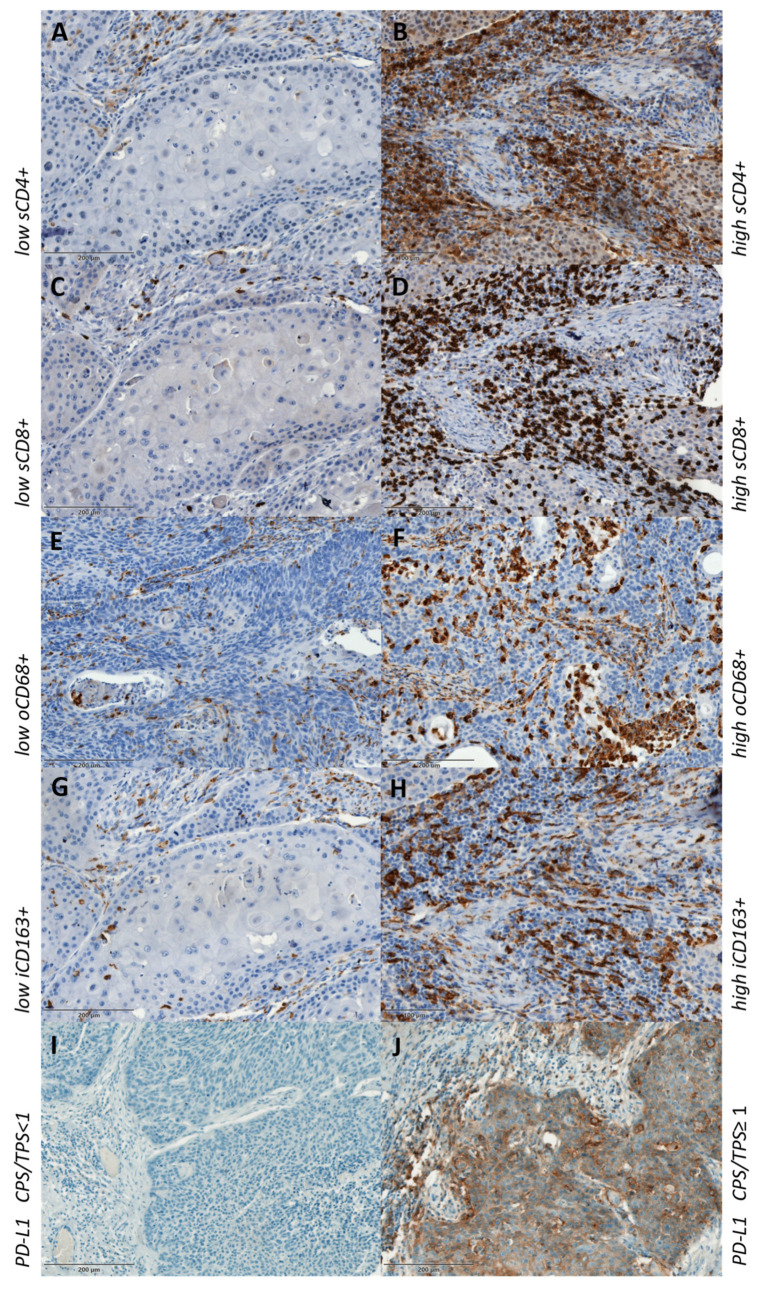
Low and high expression of immunohistochemical staining of CD4+ (**A**,**B**), CD8+ (**C**,**D**), CD68+ (**E**,**F**), CD163+ (**G**,**H**) and PD-L1-negative and -positive CPS/TPS (**I**,**J**). s—stromal; o—overall; i—intratumoral.

In the whole LSCC study group, the majority of tumors were PD-L1-positive (64.7%) when looking at the CPS score, while the PD-L1 TPS was positive in 36.3% of cases (Table 1). When comparing CPS and TPS expression between early and advance stage LSCC, we did not find a statistically significant difference (*p* = 0.363 and *p* = 0.714, respectively). However, CD8 stromal and CD68 overall expression showed statistically significant higher levels of positive cells in the advanced LSCC stage in comparison to the early stage LSCC (*p* = 0.031 and *p* = 0.027, respectively) (Table 2). Also, when looking at CD68 stromal and CD163 intratumoral expression, we found a higher level of positive cells in the advanced stage of LSCC, but it was at the level of a statistical trend (*p* = 0.059 and *p* = 0.084, respectively). This could probably reach statistical significance in the case of a larger study cohort. Figure 2 shows examples of immunohistochemical staining of the investigated markers.

### 3.3. Correlation of PD-L1 Expression with CD4, CD8, CD68 and CD163 in LSCC

In our study, PD-L1 expression evaluated as CPS and TPS showed positive correlations in comparison with CD8 and CD68 in both the intratumoral (rs = 0.202, *p* = 0.056; rs = 0.342, *p* = 0.001 for CPS and rs = 0.198, *p* = 0.003; rs = 0.311, *p* = 0.003 for TPS, respectively) and stromal (rs = 0.251, *p* = 0.017; rs = 0.259, *p* = 0.014 for CPS and rs = 0.210, *p* = 0.047; rs = 0.208, *p* = 0.049 for TPS, respectively) compartments of the LSCC group. Furthermore, there was a statistically significant positive correlation between CPS and CD163 in both intratumoral and stromal compartments (rs = 0.273, *p* = 0.008 for intratumoral and rs = 0.280, *p* = 0.007 for stromal), as well as TPS and CD163 stromal (rs = 0.257, *p* = 0.013). On the other hand, a statistically significant positive correlation between PD-L1 and CD4 was only found between CPS and CD4 stromal expression (rs = 0.269, *p* = 0.011) (Table 3). 

### 3.4. Survival Analysis and Association of PD-L1 Expression with Clinicopathological Parameters in LSCC

**Figure 3:**
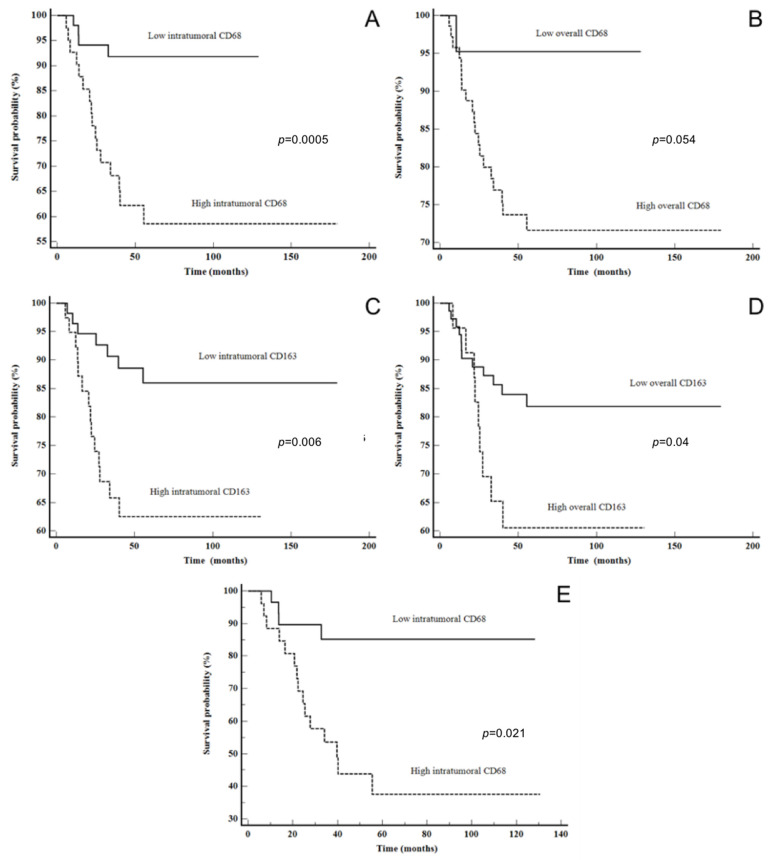
Disease-specific survival: (**A**) CD68 intratumoral in all LSCC patients, (**B**) CD68 overall in all LSCC patients, (**C**) CD163 intratumoral in all LSCC patients, (**D**) CD163 overall in all LSCC patients and (**E**) CD68 intratumoral in advanced stage LSCC.

The log-rank analysis of DSS using the Kaplan–Meier method did not show statistically significant results for CD4 intratumoral, stromal or overall expression in the whole LSCC study group. However, patients with lower CD4 stromal expression had better DSS (*p* = 0.08), and the survival plots showed a curve deviation, which suggests that the results might be significant in the case of a larger study group (Figure S1 in Supplementary Data). Similar results were obtained with CD8 intratumoral expression in the whole and advanced LSCC groups (*p* = 0.07 and 0.08, respectively), but higher expression was associated with better DSS in this case (Table 4 and Figure S1 in Supplementary Data). When looking at the whole LSCC group, patients with high CD68 and CD163 expression intratumorally (*p* = 0.0005 and *p* = 0.006, respectively) had statistically significant shorter DSS. Similar results were obtained for CD68 overall, but the results were at the level of a statistical trend (*p* = 0.054). Also, patients with higher CD163 overall had shorter DSS (*p* = 0.04). Furthermore, a shorter DSS was found in patients with high CD68 intratumoral expression, particularly when observing patients with advanced LSCC (*p* = 0.021) (Figure 3 and Table 4). Also, when we performed the overall survival (OS) analysis for all of the examined characteristics of the microenvironment, we did not find a statistically significant result (Figure S2). In the survival analysis for PD-L1 expression (evaluated as CPS and TPS), we used three different cut-offs (CPS and TPS ≥ 1 or <1, median and ROC analysis), but neither of those showed statistical significance for DSS, DFS or OS in the whole LSCC or advanced stage LSCC group. When comparing the clinical and histopathological features of LSCC with PD-L1 expression in this study, we did not find a statistically significant correlation (Table S1).

**Figure 4:**
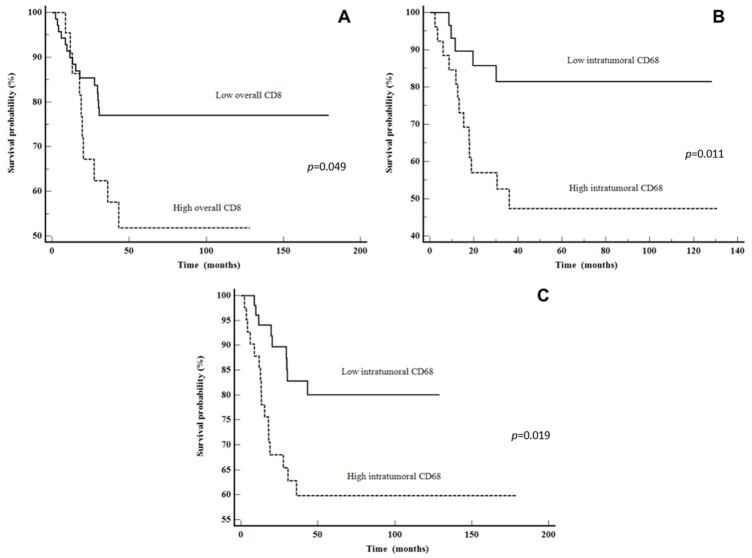
Disease-free survival: (**A**) CD8 overall in all LSCC patients, (**B**) CD68 intratumoral in all LSCC patients and (**C**) CD68 intratumoral in advanced stage LSCC patients.

The Kaplan–Meier plots illustrate a statistically shorter time to recurrence for patients with a high CD8 overall and CD68 intratumoral counts (*p* = 0.049 and *p* = 0.019, respectively). Moreover, when looking at only the advanced stage group, patients with high CD68 intratumoral had a shorter time to recurrence (*p* = 0.011). The data are shown in Figure 4. After multivariate analysis, only high CD68 intratumorally was shown to be an independent predictor of DSS (*p* = 0.0395) (Table 5).

**Figure 5:**
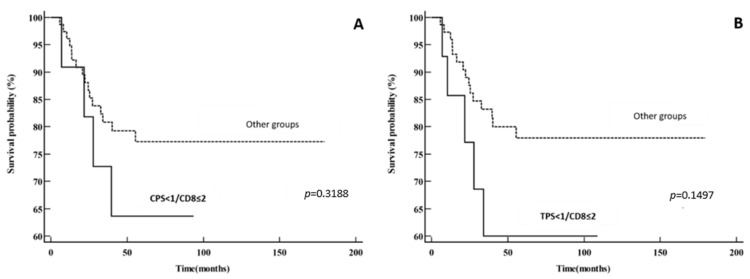
Disease-specific survival: (**A**) PD-L1^−^CD8 low vs. other groups (PD-L1^+^CD8 high, PD-L1^+^CD8 low and PD-L1^−^CD8 high) using CPS for PD-L1 expression; (**B**) PD-L1^−^CD8 low vs. other groups (PD-L1^+^CD8 high, PD-L1^+^CD8 low and PD-L1^−^CD8 high) using TPS for PD-L1 expression.

Also, we stratified patients into four groups according to PD-L1 (using both CPS and TPS) and CD8 intratumoral expression (CD8 ≤ 2% and CD8 > 2%): PD-L1^+^CD8 high, PD-L1^−^CD8 low, PD-L1^+^CD8 low and PD-L1^−^CD8 high. The group of CPS < 1/CD8 ≤ 2% had worse DSS when compared to the other groups combined (five-year DSS of 63.6% vs. 77.3%). Moreover, the group of TPS < 1/CD8 ≤ 2% had even worse survival (five-year DSS of 60% vs. 77.9%); however, statistical significance was not achieved (*p* = 0.3188 for CPS and *p* = 0.1497 for TPS). The data are shown in Figure 5.

Also, the odds ratio (OR) was calculated for the recurrence prediction in the whole LSCC group (Table 6). Statistical significance was obtained only for a high expression of CD8 overall (CD8 > 23%) and high CD68 intratumoral (CD68 > 2.7%). Patients who had high CD8 overall expression had a 3.11-times higher risk of recurrence than patients with low CD8 expression (*p* = 0.028, OR = 3.11). Similarly, patients who had high CD68 intratumoral expression had a 3.13-times higher risk of recurrence than patients with low intratumoral CD68 expression (*p* = 0.019, OR = 3.13) (Table 6). 

## 4. Discussion

It is generally known that immune-related cells play an important role in the development and progression of head and neck cancer, so it is not surprising that they have been intensively studied in recent decades. There are numerous controversies, and it is not yet fully understood the exact role of every cell component of the microenvironment in the cancer progression. In particular, immune cells comprising the microenvironment of LSCC, as well as their distribution and association with PD-L1, have not been well studied.

PD-L1, also known as B7-H1, is a type I transmembrane glycoprotein that plays a crucial role in tumor immunity and is widely expressed in immune, epithelial and tumor cells [20]. A high level of PD-L1 expression in tumor cells allows them to evade the host immune response and favors tumor progression [21]. On the other hand, PD-L1 expression is regulated by IFN-γ secretion from T lymphocytes [21]. In this case, tumors that have high T lymphocyte infiltration will subsequently have a better immune response, which results in a higher expression of PD-L1 in tumor cells and a better outcome of disease. Consequently, PD-L1 can be seen as both a biomarker of constant immune pressure and as an immune response inhibitor [22].

In our study, 64.7% of tumors had a positive PD-L1 CPS score, but we did not find the impact of PD-L1 expression on survival. Similar results were obtained in other studies [23,24,25]. Hirshoren et al. included 26 oral squamous cell cancer and 10 LSCC patients and did not find the impact of CPS on overall (OS) [23], disease-specific or progression-free survival (PFS) (*p* = 0.45, *p* = 0.31 and *p* = 0.88, respectively). Batur et al. included 52 LSCC patients and also did not find an association between the CPS score and OS (*p* = 0.413) [24]. Wusiman et al. included 119 patients with HNSCC and did not find a correlation between the CPS score and OS or PFS using CPS cut-off values of 1 or 20 [25]. However, the study cohort included only 40 LSCC patients, and survival analysis was done on the whole HNSCC group and not on the LSCC subgroup only. 

Tumor-infiltrating lymphocytes (TILs) are shown to have prognostic significance in cancer patients [19]. Among immune cells, CD8+ lymphocytes stand out due to their antitumor response, which leads to a better prognosis of disease with longer DSF and improved overall survival (OS) in different cancer types [13,14,15]. The reduced number of TILs is the result of PD-L1 to PD-1 binding, which, in turn, causes effector CD8+ T cells in tumor tissue to undergo cell death. 

According to the results from this study, CD8+ T cells in both compartments, intratumoral and stromal, positively correlated with PD-L1 expression, evaluated as TPS. Also, there was a statistically significant positive correlation between the stromal compartment of CD8+ T cells and CPS. Furthermore, a higher CD8+ T cell intratumoral distribution (CD8+ ≤ 2% cut-off) was associated with better DSS (*p* = 0.07 for the whole and *p* = 0.08 for the advanced LSCC group), which is in agreement with previous studies [10]. Also, in this study, the high level of overall CD8+ T lymphocytes infiltration (CD8+ > 23% cut-off) was found to be a good predictor for LSCC recurrence (*p* = 0.028, OR = 3.11) and was associated with worse DFS. Those two results from our study, of high CD8 infiltration and better DSS but, at the same time, worse DFS, are opposing, and this can be due to T cell exhaustion. The current studies that are examining the prognostic impact of CD8+ T cell infiltration in cancer are inconsistent, indicating the heterogeneity of intratumoral cytotoxic T lymphocytes. Opposite the conventional activated CD8+ T cells that act as immune effectors, persistent antigen stimulation may lead to a dysfunctional state called T cell exhaustion [26]. Some studies have shown that PD-L1 expression significantly correlates with the presence of PD-1+ TILs [27]. PD-1, a crucial marker to inhibit T cell activation, is typically overexpressed on exhausted T cells, so abundant PD-1+CD8+ TILs are associated with a worse prognosis and impaired antitumor response in many malignancies [28,29,30]. The findings from this study, where a higher CD8+ infiltration seems in favor of recurrence and worse DFS in LSCC, are in accordance with the study of Ahmadvand S et al., where PD-1+ CD8+ T cells were expected to be in a hyporesponsive state, but at the same time, they were representative of antitumor immune response formation [27]. 

CD68 is the most widely recognized generic marker for all macrophages, whereas CD163 is the most extensively employed marker for differentiating M2 polarized macrophages. Macrophages have the potential to promote tumor metastasis and proliferation through various mechanisms. They have the ability to promote angiogenesis and augment the invasion, motility and intravasation of tumor cells within the primary tumor [31]. It is common for the CD163+ subset of macrophages to attract effector T cells that are unable to develop a protective antitumor immune response. This is accomplished by the release of interleukins (IL-4, IL-13 and IL-10) and other immunosuppressive cytokines [10]. Using a variety of different ways, macrophages can suppress cytotoxic T cell responses. According to Kuang et al., macrophages are responsible for the production of IL-10, which, in turn, causes monocytes to express PD-L1 and decreases cytotoxic T cell responses [32]. In human ovarian cancer, chemokine, known as CCL22, is produced by macrophages. This chemokine controls the inflow of regulatory T cells, which are responsible for suppressing cytotoxic T cell responses [32].

The ongoing therapeutic challenge is to boost the activation of antitumoral activities of macrophages while blocking their trophic phenotypes and immunosuppressive behaviors [33]. Targeting these cells may elicit a more pronounced response due to their extensive participation in carcinogenesis; they may exert their influence at various stages along the oncogenesis pathway within a single tumor. Additionally, it has the potential to serve as a therapeutic intervention for various malignant tumor types that contain CD163+ TAMs. Given that the five-year survival rate of advanced LSCC is only 50%, it is critical to investigate CD68+ and CD163+ TAMs as a potential therapeutic tool for LSCC.

Our study showed that a higher level of CD68 (>2.7% cut-off) and CD163 (>2% cut-off) intratumoral (*p* = 0.0005 and *p* = 0.006, respectively) was associated with a worse DSS. Moreover, higher CD68 intratumoral infiltration was also found to be associated with worse DFS and was established as a good predictor for LSCC recurrence (*p* = 0.019, OR = 3.13). Furthermore, the multivariate analysis for our whole LSCC study group showed high CD68 intratumoral expression to be an independent predictive marker of DSS. These results can be explained with the aforementioned protumoral and immunosuppressive features of macrophages. The results of our study indicate that the level of infiltration with CD68 and CD163 macrophages is a significant predictive factor for patients with LSCC.

Teng et al. proposed a model that classified the tumor microenvironment into four distinct categories based on the PD-L1 expression and density of the TILs: type I (PD-L1^+^TILs^+^), type II (PD-L1^−^TILs^−^), type III (PD-L1^+^TILs^−^) and type IV (PD-L1^−^TILs^+^). Individuals diagnosed with type I were shown to have the highest likelihood of experiencing positive outcomes with anti-PD-1/PD-L1 blocking treatment [34]. Kim et al. also highlighted the significance of evaluating both PD-L1 expression and TILs when choosing patients for anti-PD-1/PD-L1 therapy, as it helps to identify those who are more likely to react positively to the treatment [35]. In our study, we did not find a significant difference in DSS or DFS between these four groups. However, when comparing the CPS < 1/CD8 ≤ 2% group and TPS < 1/CD8 ≤ 2% group with the other groups, we found that these groups had worse survival (*p* = 0.3188 and *p* = 0.1497, respectively). These groups probably would not have much benefit in only anti-PD-1/PD-L1 therapy, given the lack of preexisting CD8+ T lymphocytes. According to Teng, a large number (40%) of melanoma patients have the PD-L1^−^TILs^−^ histologic type [34]. Regardless of treatment intervention, this group of patients has a very unfavorable prognosis. However, establishing this at baseline would aid in determining whether to administer combination immunotherapies, which have the potential to reverse this condition in certain instances. Further research on this topic is certainly warranted, because 40–45% of patients fail to respond to the checkpoint anti-PD-1/PD-L1 inhibitory therapy [27]. Therefore, to determine which LSCC patients will benefit from anti-PD-1/PD-L1 inhibitors, it is crucial to elucidate the relationship between PD-L1 expression, immune cell distribution and prognosis in laryngeal patients. 

The main strength of this study is the uniformity of the patient cohort, all of whom underwent primary laryngeal surgery followed by chemo- or radiation therapy. Frequently, HNSCC studies include a heterogeneous population of patients with tumors from different anatomical sites. Only definitive surgical specimens of LSCC were evaluated, while small, probatory biopsies were not included. In this study we used the PD-L1 antibody, clone SP263, from Ventana, Tucson, SAD, due to its efficacy and preserved expression in prolonged room temperature section storage in formalin-fixed paraffin-embedded tissue (FFPE), especially when the FFPE blocks were older than 3 years, unlike clone 22C3, which, when used, could lead to underestimation of the PD-L1 status, particularly in this setting [36,37]. De Ruiter et al. showed that SP263 stained a higher percentage of cells when using the CPS or TPS; however, they did not take the length of storage of the tissue into consideration, which might be the reason for a discrepant result when using clone 22C3 [38]. Furthermore, we used both CPS and TPS as PD-L1 scoring systems; unfortunately, there is no agreement in the literature on cut-offs, so we tried to used different options, but neither one showed statistical significance. Also, CD68 and CD163 were employed as the most frequently used markers for all and M2 macrophages, respectively [10]. We examined CD4, CD8, CD68 and CD163 cells for analysis of the tumor microenvironment in both intratumoral and stromal compartments, as well as the overall number of tumor microenvironment cells. This study also included the relatively same number of patients with similar proportions of the early and advanced stages of LSCC with a long median follow-up time, while the primary limitation of the research was the retrospective aspect of the study with a relatively small number of examined cases and the use of TMAs. However, to compensate for using TMA-stained slides, we used three to four 1 mm tissue cores and serial sections of the same TMA cores.

## 5. Conclusions

In conclusion, our research demonstrated a significant correlation between PD-L1 expression and tumor microenvironment cells in LSCC patients. Furthermore, CD68 and CD163 macrophages were found to be a possible significant predictive factor for patients with LSCC. To clarify this link and identify LSCC patients who might benefit the most from anti-PD-1/PD-L1 therapy, larger studies are required.

## Figures and Tables

**Table 1:** Demographic features of the LSCC group.

Characteristic of Patients with LSCC N = 102		Number of Patients (%)
Age (years); median (range)		63.0 (43.9–83.6)
Sex	Female	7 (6.9)
	Male	95 (93.1)
Smoking	No	25 (24.5)
	Yes	77 (75.5)
Alcohol	No	54 (52.9)
	Yes	48 (47.1)
T classification	1	26 (25.5)
	2	17 (16.7)
	3	40 (39.2)
	4	19 (18.6)
Clinical stage	I	26 (25.5)
	II	18 (17.6)
	III	34 (33.3)
	IV	24 (23.5)
N classification	0	87 (85.3)
	1	10 (9.8)
	2	5 (4.9)
Localization	Supraglottic	9 (8.8)
	Glottic	72 (70.6)
	Subglottic	3 (2.9)
	Transglottic	18 (17.6)
Histological grade	G1	21 (21.4)
	G2	56 (54.4)
	G3	19 (18.4)
	Unknown	6 (5.8)
Lymph vessel invasion	No	46 (45.6)
	Yes	39 (37.9)
	Unknown	17 (16.5)
Blood vessel invasion	No	46 (45.6)
	Yes	38 (37.3)
	Unknown	17 (17.1)
Perineural invasion	No	73 (71.8)
	Yes	11 (10.7)
	Unknown	18 (17.5)
Resection margins	R0	90 (88.2)
	R1	12 (11.8)
Recurrence	No	76 (74.5)
	Yes	26 (25.5)
CPS	<1	27 (26.5)
	≥1	66 (64.7)
	Unknown	9 (8.8)
TPS	<1	56 (54.9)
	≥1	37 (36.3)
	Unknown	9 (8.8)
Months to recurrence		
Median (Range)		15.3 (2.3–43.3)
Follow-up (months)		
Median (Range)		56.2 (0.1–179.5)
Died of the disease N (%)		21 (20.4)
Occurrence of second cancer N (%)		26 (25.2)

Abbreviations: LSCC—laryngeal squamous cell cancer; CPS—combined positive score; TPS—tumor proportion score.

**Table 2:** Comparison of the expression of PD-L1, CD4, CD8, CD68 and CD163 between the early and advanced stages of LSCC.

Variable, Median (Range)		LSCC Early	LSCC Advanced	*p*-Value
N = 102		N = 44	N = 58	
CD4	intratumoral	1.5 (0.0–20.0)	1.0 (0.0–6.7)	0.619 ^¶^
	stromal	8.0 (0.0–25.7)	10.0 (0.0–40.3)	0.263 ^¶^
	overall	10.0 (0.0–50.0)	10.5 (0.0–53.3)	0.639 ^¶^
CD8	intratumoral	2.5 (0.0–18.3)	3.0 (0.0–30.0)	0.846 ^¶^
	stromal	5.3 (0.0–25.3)	10.0 (0.0–37.3)	**0.031 ^¶^**
	overall	8.0 (0.2–40.0)	13.5 (0.5–60.0)	0.109 ^¶^
CD68	intratumoral	2.0 (0.0–9.3)	2.5 (0.0–10.0)	0.280 ^¶^
	stromal	7.0 (1.5–30.0)	8.5 (0.1–27.5)	0.059 ^¶^
	overall	8.0 (1.0–45.0)	12 (0.3–50.0)	**0.027 ^¶^**
CD163	intratumoral	1.25 (0.0–9.0)	2.0 (0.0–12.0)	0.084 ^¶^
	stromal	15.0 (0.0–50.0)	13.7 (0.0–35.0)	0.348 ^¶^
	overall	13.5 (0.0–50.0)	15.8 (0.0–50.0)	0.165 ^¶^
TPS		3.9 (0.0–76.7)	2.7 (0.0–72.5)	0.714 ^¶^
CPS	<1	13 (29.5)	14 (25.5)	0.363 ^§^
	≥1	25 (56.9)	41 (74.5)	
	NA	6 (13.6)	3 (5.2)	

^¶^ Mann–Whitney test; ^§^ Fisher’s exact test. Abbreviations: CPS—combined positive score; TPS—tumor proportion score; bold *p* value is statistically significant.

**Table 3:** Correlation of CPS and TPS with intratumoral and stromal CD4-, CD8-, CD68- and CD163-positive cells in LSCC.

			CD4	CD8	CD68	CD163
Intratumoral	CPS	r_s_	0.194	0.202	**0.342**	**0.273**
		*p*	0.069	0.056	**0.001**	**0.008**
	TPS	r_s_	0.124	**0.198**	**0.311**	0.182
		*p*	0.249	**0.003**	**0.003**	0.081
			**CD4**	**CD8**	**CD68**	**CD163**
Stromal	CPS	r_s_	**0.269**	**0.251**	**0.259**	**0.280**
		*p*	**0.011**	**0.017**	**0.014**	**0.007**
	TPS	r_s_	0.182	**0.210**	**0.208**	**0.257**
		*p*	0.088	**0.047**	**0.049**	**0.013**

Abbreviations: LSCC—laryngeal squamous cell cancer; CPS—combined positive score; TPS—tumor proportion score, bold values are statistically significant.

**Table 4:** Univariate survival analysis for the whole and advanced LSCC.

	Variables (Cut-Off; %)	N	Died from Disease	Survival (%)	Mean ± SD	95% CI	χ^2^	Log-Rank Test (*p*)
Whole LSCC Group	CD4 intratumoral (>0.4)							
	Low	28	3	10.71	160.7 ± 10.16	140.82–180.65	2.55	0.11
	High	63	16	25.40	99.5 ± 6.33	87.1–111.92		
	CD4 stromal (>6.5)							
	Low	30	3	10	162.06 ± 9.52	143.39–180.73	3.04	0.08
	High	61	16	26.3	99.52 ± 6.6	86.57–112.46		
	CD4 overall (>11.5)							
	Low	51	8	15.69	151.37 ± 9.07	133.58–169.15	1.46	0.23
	High	40	11	27.5	99.29 ± 7.99	83.62–114.97		
Advanced LSCC Group	CD4 intratumoral (>0.4)							
	Low	17	3	17.65	108.5 ± 11.3	86,33–130,66	2.62	0.10
	High	37	15	40.54	82.11 ± 9.12	64.24–99.99		
	CD4 stromal (>6.5)							
	Low	15	3	20	96.72 ± 11	75.16–118.28	1.35	0.25
	High	39	15	38.46	84.91 ± 9.06	67.16–102.66		
	CD4 overall (>11.5)							
	Low	29	8	27.59	93.66 ± 10.37	73.34–113.98	0.6	0.44
	High	25	10	40	84.27 ± 11.12	62.48–106.06		
Whole LSCC Group	CD8 intratumoral (≤2)							
	Low	40	12	30	128.67 ± 12.2	104.75–152.59	3.28	0.07
	High	53	8	15.1	113.04 ± 5.68	101.9–124.17		
	CD8 stromal (>12)							
	Low	60	11	18.33	147.53 ± 8.67	130.54–164.51	0.59	0.44
	High	33	9	27.27	98.73 ± 8.32	82.43–115.03		
	CD8 overall (>23)							
	Low	71	13	18.31	147.27 ± 8.02	131.55–163	1.07	0.3
	High	22	7	31.82	95.52 ± 10.27	75.4–115.64		
Advanced LSCC Group	CD8 intratumoral (≤2)							
	Low	24	11	45.83	69.17 ± 10.63	48,33–90.01	3.04	0.08
	High	30	8	26.67	99.3 ± 9.33	81.01–117.59		
	CD8 stromal (>12)							
	Low	31	10	32.26	91.54 ± 10.09	71.76–111.32	0.08	0.77
	High	23	9	39.13	84.26 ± 11.08	62.54–105.98		
	CD8 overall (>23)							
	Low	39	12	30.77	92.4 ± 9.03	74.71–110-1	0.48	0.49
	High	15	7	46.67	80.22 ± 13.33	54.1–106.34		
Whole LSCC Group	CD68 intratumoral (>2.7)							
	Low	53	4	7.55	119.94 ± 4.35	111.42–128.46	11.97	0.0005
	High	41	16	39.02	115.39 ± 12.48	90.93–139.84		
	CD68 stromal (>4.5)							
	Low	24	2	8.33	118.17 ± 6.85	104.74–131.59	2.67	0.1
	High	70	18	25.71	135.92 ± 8.79	118.69–153.15		
	CD68 overall (>5.5)							
	Low	23	1	4.35	122.69 ± 5.48	111.95–133.43	3.7	0.054
	High	71	19	26.76	135.37 ± 8.64	118.44–152.3		
Advanced LSCC Group	CD68 intratumoral (>2.7)							
	Low	29	4	13.79	112.04 ± 7.54	97.27–126.82	9.44	0.021
	High	26	15	57.69	65.3 ± 10.8	44.12–86.47		
	CD68 stromal (>4.5)							
	Low	11	2	18.18	107.01 ± 13.59	80.37–133.65	1.34	0.25
	High	44	17	38.64	84.61 ± 8.58	67.79–101.42		
	CD68 overall (>5.5)							
	Low	9	1	11.11	115.2 ± 12.35	90.99–139.41	1.57	0.21
	High	46	18	39.13	85.18 ± 8.25	69.01–101.35		
Whole LSCC Group	CD163 intratumoral (>2)							
	Low	58	7	12.07	158.33 ± 7.46	143.71–172.96	7.6	0.006
	High	39	14	35.9	89.6 ± 8.75	72.46–106.74		
	CD163 stromal (>18.3)							
	Low	64	11	17.99	148.88 ± 8.37	132.48–165.27	1.16	0.28
	High	33	10	30.3	98.57 ± 8.49	81.93–115.21		
	CD163 overall (>27.3)							
	Low	74	12	16.22	151.12 ± 7.46	136.5–165.75	4.2	0.04
	High	23	9	39.13	88.73 ± 10.93	67.32–110.15		
Advanced LSCC Group	CD163 intratumoral (>2)							
	Low	29	7	24.14	83.84 ± 8.07	68.03–99.65	2.21	0.14
	High	28	13	46.43	78.9 ± 10.52	58.29–99.51		
	CD163 stromal (>18.3)							
	Low	36	10	27.78	94.02 ± 9.15	76.08–111.96	0.99	0.32
	High	21	10	47.62	80.07 ± 11.61	57.3–102.83		
	CD163 overall (>27.3)							
	Low	40	11	27.5	78.28 ± 8.5	78.28–111.58	2.13	0.14
	High	17	9	52.94	73.67 ± 13.05	48.08–99.26		

Abbreviations: LSCC—laryngeal squamous cell cancer.

**Table 5:** Multivariate survival analysis for the whole LSCC.

Disease-Specific Survival			
Variables (Cut-Off; %)	HR	95% CI	*p*-Value
CD68 intratumoral (>2.7)	3.66	1.06–12.57	0.0395
CD68 overall (>5.5)	2.36	0.28–20.03	0.4319
CD163 intratumoral (>2)	1.47	0.51–4.21	0.4727
CD163 overall (>27.3)	1.20	0.46–3.15	0.7087
**Disease-free survival**			
CD8 overall (>23)	1.68	0.73–3.91	0.2249
CD68 intratumoral (>2.7)	2.20	0.95–5.10	0.0665

Abbreviations: LSCC—laryngeal squamous cell cancer; HR—hazards ratio; CI—confidence interval.

**Table 6:** Predictors for the recurrence of whole LSCC.

Predictors; Cut-Off (%)		OR *	95% CI	*p*-Value	AUC
CD4	intratumoral > 0.4	2.59	0.79–8.49	0.116	0.590
	stromal > 6.5	1.16	0.42–3.24	0.765	0.517
	overall > 11.5	1.56	0.60–4.03	0.360	0.555
CD8	intratumoral ≤ 2	0.76	0.30–1.91	0.556	0.534
	stromal > 12	2.07	0.81–5.28	0.129	0.586
	overall > 23	**3.11**	**1.13–8.58**	**0.028**	**0.612**
CD68	intratumoral > 2.7	**3.13**	**1.21–8.11**	**0.019**	**0.639**
	stromal > 4.5	2.14	0.65–7.04	0.209	0.565
	overall > 5.5	2.99	0.80–11.14	0.102	0.585
CD163	intratumoral > 2	1.73	0.69–4.29	0.236	0.567
	stromal > 18.3	1.63	0.65–4.13	0.299	0.557
	overall > 27.3	2.16	0.79–5.84	0.131	0.574
CPS (<1, ≥1)		1.31	0.46–3.78	0.614	0.527
CPS ≤ 7.5 (median)		1.68	0.41–2.83	0.467	0.541
TPS ≤ 2.7 (median)		1.03	0.41–2.61	0.951	0.504

* Odds ratio was calculated based on cut-offs and consequently classified into higher and lower values. Abbreviations: LSCC—laryngeal squamous cell cancer; CPS—combined positive score; TPS—tumor proportion score; bold are statistically significant results.

## Data Availability

The datasets analyzed during the current study are available from the corresponding author upon reasonable request.

## Supplementary Materials

The following supporting information can be downloaded at https://www.mdpi.com/article/10.3390/cancers16152645/s1. Figure S1: Disease specific survival; A—CD4 stromal in all LSCC patients, B—CD8 intratumoral in all LSCC patients, C—CD8 intratumoral in advanced LSCC patients; Figure S2: Overall survival; A—CD8 intratumoral (iCD8+) in all LSCC patients, B—CD8 overall (oCD8+) in all LSCC patients, C—CD68 intratumoral (iCD8+) in all LSCC patients, D—CD68 stromal (sCD68+) in all LSCC patients; Table S1: Correlation of CPS with clinicopathological features of the LSCC group.
