# Supplementary material for: Significance of PD-L1 and Tumor Microenvironment in Laryngeal Squamous Cell Cancer
Source: Cancers (Basel). 2024 Jul 25;16(15):2645. doi: 10.3390/cancers16152645 (PMC11311265; doi:10.3390/cancers16152645)
Supplement: Supplementary file 1 [file cancers-16-02645-s001.zip › cancers-3081850-supplementary.pdf]

Supplementary Materials

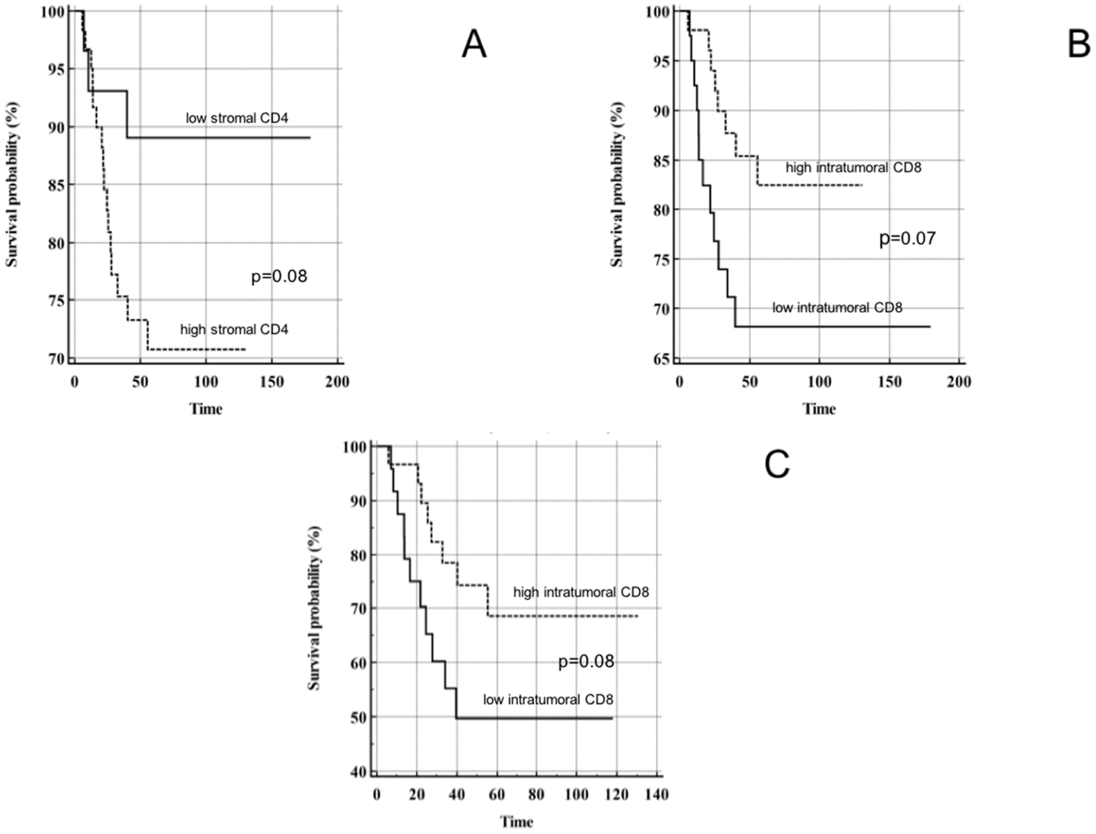

Figure S1. Disease specific survival; A – CD4 stromal in all LSCC patients, B – CD8 intratumoral in all LSCC patients, C – CD8 intratumoral in advanced LSCC patients

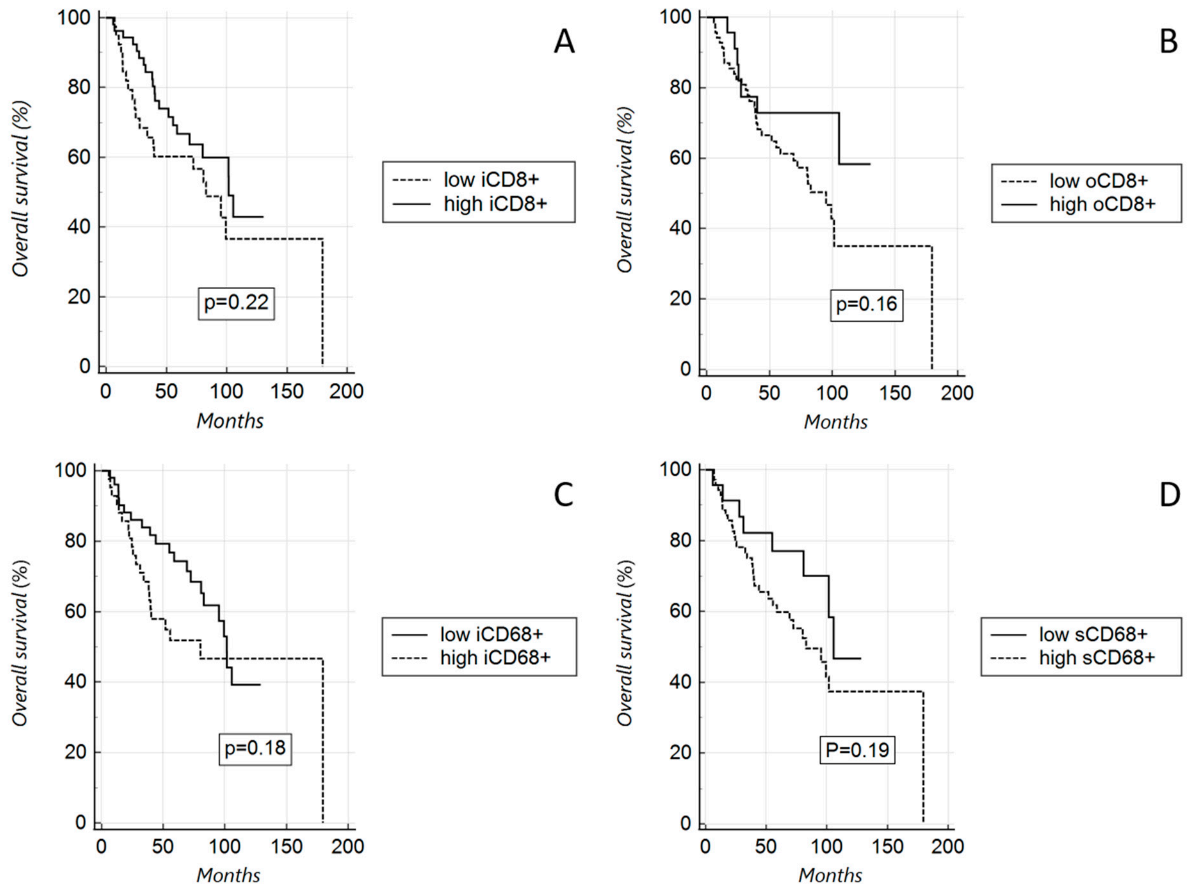

Figure S2. Overall survival; A – CD8 intratumoral (iCD8+) in all LSCC patients, B – CD8 overall (oCD8+) in all LSCC patients, C – CD68 intratumoral (iCD8+) in all LSCC patients, D – CD68 stromal (sCD68+) in all LSCC patients

Table S1. Correlation of CPS with clinicopathological features of the LSCC group

N=93		CPS		
		**<1 (N=27)**	**≥ 1 (N=66)**	p value
**Smoking**				
	No	7 (25.9)	16 (24.2)	P=1.00§
	Yes	20 (74.1)	50 (75.8)	
**Alcohol**				
	No	15 (55.6)	32 (48.5)	P=0.649§
	Yes	12 (44.4)	34 (51.5)	
**Histological grade**				
	G1	4 (14.8)	13 (20.9)	χ^2 =0.888 DF=2 P=0.641
	G2	17 (63.0)	36 (53.7)	
	G3	4 (14.8)	13 (20.9)	
	unknown	2 (7.4)	4 (4.5)	
**T classification**				
T1		6 (22.2)	15 (22.4)	χ^2 =2.766 DF=4 P=0.598
T2		7 (25.9)	9 (13.4)	
T3		10 (37.0)	27 (41.8)	
T4		4 (14.8)	15 (22.4)	
**N classification**				
	N0	25 (92.6)	55 (83.3)	P=0.335§
	N1 and N2	2 (7.4)	11 (16.7)	
**Clinical stage**				
	1	6 (22.2)	15 (22.4)	χ^2 =2.245 DF=4 P=0.691
	2	7 (25.9)	10 (14.9)	
	3	9 (33.3)	23 (35.8)	
	4	5 (18.5)	18 (26.9)	
**Lymph vessel invasion**				
	Absent	13 (48.1)	28 (43.3)	P=0.806§
	Present	10 (37.0)	27 (40.3)	
	Unknown	4 (14.8)	11 (16.4)	
**Blood vessel invasion**				
	Absent	13 (48.1)	29 (44.8)	P=0.807§
	Present	10 (37.0)	26 (38.8)	
	Unknown	4 (14.8)	11 (16.4)	
**Perineural invasion**				
	Absent	19 (70.4)	48(73.1)	P=0.474§
	Present	4 (14.8)	6 (9.0)	
	Unknown	4 (14.8)	12 (17.9)	
**Recurrence**				
	No	21 (77.8)	48 (72.7)	P=0.795§
	Yes	6 (22.2)	18 (27.3)	
**Months to recurrence, median (range)**		22.6 (5.97-30.53)	13.1 (2.27-36.07)	P=0.274¶
**Died of the disease**				
	No	21 (77.8)	52 (78.8)	P=1.00§
	Yes	6 (22.2)	14 (21.2)	
**Follow up in months, median (range)**		72.3 (0.1-129.0)	47.2 (5.8-179.5)	P=0.324¶

¶Mann-Whitney test; §Fisher's exact test, ±Chi-squared test

Abbreviations: LSCC – laryngeal squamous cell cancer; CPS – combined positive score
